# Conserved Oligomeric Golgi (COG) Complex Proteins Facilitate Orthopoxvirus Entry, Fusion and Spread

**DOI:** 10.3390/v12070707

**Published:** 2020-06-30

**Authors:** Susan Realegeno, Lalita Priyamvada, Amrita Kumar, Jessica B. Blackburn, Claire Hartloge, Andreas S. Puschnik, Suryaprakash Sambhara, Victoria A. Olson, Jan E. Carette, Vladimir Lupashin, Panayampalli Subbian Satheshkumar

**Affiliations:** 1 Poxvirus and Rabies Branch, Centers for Disease Control and Prevention, Atlanta, GA 3033, USA; srealegeno@gmail.com (S.R.); odk7@cdc.gov (L.P.); moo0@cdc.gov (C.H.); vao9@cdc.gov (V.A.O.); 2 Influenza Division, Centers for Disease Control and Prevention, Atlanta, GA 3033, USA; vtf1@cdc.gov (A.K.); zao1@cdc.gov (S.S.); 3 Department of Physiology and Biophysics, University of Arkansas for Medical Sciences, Little Rock, AR 72205, USA; jessica.b.blackburn@vumc.org (J.B.B.); vvlupashin@uams.edu (V.L.); 4 Department of Microbiology and Immunology, Stanford University, Stanford, CA 94035, USA; andreas.puschnik@czbiohub.org (A.S.P.); carette@stanford.edu (J.E.C.)

**Keywords:** COG complex, vaccinia, monkeypox, viral attachment, viral entry, glycosylation

## Abstract

Although orthopoxviruses (OPXV) are known to encode a majority of the genes required for replication in host cells, genome-wide genetic screens have revealed that several host pathways are indispensable for OPXV infection. Through a haploid genetic screen, we previously identified several host genes required for monkeypox virus (MPXV) infection, including the individual genes that form the conserved oligomeric Golgi (COG) complex. The COG complex is an eight-protein (COG1–COG8) vesicle tethering complex important for regulating membrane trafficking, glycosylation enzymes, and maintaining Golgi structure. In this study, we investigated the role of the COG complex in OPXV infection using cell lines with individual COG gene knockout (KO) mutations. COG KO cells infected with MPXV and vaccinia virus (VACV) produced small plaques and a lower virus yield compared to wild type (WT) cells. In cells where the KO phenotype was reversed using a rescue plasmid, the size of virus plaques increased demonstrating a direct link between the decrease in viral spread and the KO of COG genes. KO cells infected with VACV displayed lower levels of viral fusion and entry compared to WT suggesting that the COG complex is important for early events in OPXV infection. Additionally, fewer actin tails were observed in VACV-infected KO cells compared to WT. Since COG complex proteins are required for cellular trafficking of glycosylated membrane proteins, the disruption of this process due to lack of individual COG complex proteins may potentially impair the virus-cell interactions required for viral entry and egress. These data validate that the COG complex previously identified in our genetic screens plays a role in OPXV infection.

## 1. Introduction

The orthopoxvirus (OPXV) genus includes several viruses that can infect humans, such as variola virus (the causative agent of eradicated smallpox), monkeypox virus (an emerging zoonotic infectious pathogen) and vaccinia virus (the prototypic poxvirus member extensively used in research studies and vaccines). Poxviruses are dsDNA-enveloped viruses with relatively large genomes that encode a large proportion of the genes required for entry, replication, and assembly. There are two infectious forms, the mature virus (MV) and the extracellular virus (EV). Infection begins with viral entry, a multistep process that includes virus attachment to the host cell, fusion of viral and host membranes, and core entry into the cytoplasm. These early events in infection can vary depending on the infectious form of the virus, the virus strain, and the host target cell type, among other factors [1,2]. The initial attachment of virus to cells involves viral proteins that target host cell glycosaminoglyans (GAGs) or laminins [3,4,5]. Once EV binding occurs, the outer membrane makes way for the entry fusion complex (EFC) on the MV membrane to interact with host membrane proteins, leading to viral fusion [6,7]. This is followed by the release of viral cores into the cytoplasm where virus replication occurs.

Replication occurs exclusively in the cytoplasm in viral cytoplasmic factories resulting in the formation of both MV and EV particles. MVs are intracellular virions with a single membrane that are released only during cell lysis. A small percentage of MVs translocate to the Golgi and gain an additional double membrane [8,9,10]. These intracellular triple-wrapped viruses then migrate to the periphery, fuse with the plasma membrane and are released from the cell as EVs [11,12,13]. Although a large proportion of the proteins required for entry, replication, and assembly are encoded by the viral genome, the successful completion of the OPXV life cycle requires a number of host proteins and pathways [14,15,16,17].

One such example is the Golgi-associated retrograde protein (GARP) complex, a multi-subunit tethering factor important for Golgi retrograde transport, which has a demonstrated impact on EV formation and viral spread [16,17]. We and others have also identified additional Golgi transport genes including those belonging to the COG complex. Similar to the GARP complex, the COG complex is also a multi-subunit tethering complex functioning at the Golgi apparatus. It is an important regulator of intra-Golgi retrograde trafficking that is needed for proper recycling of glycosylation enzymes and for maintaining Golgi structure [18,19,20]. The COG complex is an eight heteromeric subunit complex made up of two lobes. Lobe A is comprised of subunits COG1–4 and Lobe B is comprised of subunits COG5–8, and the two lobes are associated through the interactions of COG1 and COG8 [21]. Studies have demonstrated that several intracellular pathogens such as HIV-1, *Chlamydia trachomatis*, and *Brucella abortus* exploit the COG complex to complete their infectious life cycle [22,23,24]. Additionally, genes from both Lobe A and Lobe B have been shown to play an important role in poxvirus infection [14,16,17,25]. In particular, our haploid genetic screen previously identified COG3, COG4, COG7 and COG8 as host factors that may be involved in monkeypox virus (MPXV) infection [16].

Here, we investigated the role of each of the COG complex subunits in OPXV infection using COG1–8 knockout cell lines. Our data demonstrated the requirement of COG complex proteins for the fusion and entry steps of vaccinia virus (VACV) infection. We also showed that the absence of COG proteins caused a decrease in the number of actin tails in infected cells, and decreased viral spread, as evidenced by smaller VACV and MPXV plaques produced in COG KO cells compared to wildtype (WT) cells. These data confirm the results of our haploid genetic screen and provide insights on the specific steps of the OPXV life cycle that are impacted by the presence or absence of COG complex genes.

## 2. Materials and Methods

### 2.1. Cells and Viruses

HEK 293T and HEK293T COG subunit knock out (KO) cell lines were cultured in DMEM/F12 50/50 media (Thermo Scientific, Waltham, MA, USA) with 10% fetal bovine serum (FBS) (Atlanta Biologicals, Oakwood, USA) in the presence of 10 units/mL of penicillin and 100 µg/mL of streptomycin (pen/strep). The COG KO cell lines are stable knockouts that were previously constructed using the CRISPR/Cas9 method and characterized as described by Blackburn et al. [20,26]. In addition, rescued COG4 and COG7 KO cell lines were constructed by stably transfecting the respective COG subunit gene fused with GFP, COG4-GFP and COG7-2 × GFP respectively, as previously described [20,27]. Rescued cells were cultured in DMEM/F12 50/50 media supplemented with 10% FBS, pen/strep, and 10 µg/mL of blasticidin.

The viruses used in the study include MPXV West African (MPXV WA) strain MPXV-USA-2003-044, VACV Western Reserve (WR), and recombinant VACV WR expressing firefly luciferase (VACV WR-LUC). All virus infections were performed using DMEM supplemented with 2% FBS (DMEM-2) as culture media.

### 2.2. Immunostaining

HEK293T wild type (WT) and COG KO cells were seeded overnight in 12-well plates for 80–100% confluency and infected with MPXV WA or VACV WR at MOI 0.01. An overlay of DMEM-2 containing 0.05% methylcellulose was added to the cells and the plates were incubated for 72 h at 37 °C. The infected cells were fixed with a 1:1 acetone-methanol solution for 15 min and washed with PBS. Cells were stained with rabbit anti-vaccinia antibody (Virostat, Inc., Portland, ME, USA) diluted 1:1000 in PBS containing 2% FBS (PBS-2) for 1 h at room temperature (RT) on a rocker. After washing with PBS, an HRP-conjugated donkey anti-rabbit antibody (Jackson ImmunoResearch Laboratories, West Grove, PA, USA) diluted 1:1000 in PBS-2 was added to cells for 1 h at RT. Cells were washed twice with PBS. The substrate was prepared by adding a saturated o-dianisidine-ethanol solution to 0.03% H_2_O_2_. The substrate was added to the cells for 5–10 min at RT until the foci could be visualized and imaged, and subsequently washed away with water to stop the reaction. Approximately 40 foci from each cell type were analyzed using ImageJ software to measure average focus area per condition. Values for each KO cell type were compared to WT for statistical significance.

### 2.3. Multi-Step Infections

WT and KO cells were infected with MPXV at MOI 0.01. Supernatants and cell monolayers were collected at 0, 24, 48 and 72 h and frozen until use. Virus yield of supernatant and cell monolayer samples from the multi-step infections were determined by plaque assay. Each cell type and time point were tested using 2–3 replicates.

### 2.4. Plaque Assays

Samples were serially diluted in DMEM-2 and added to BSC-40 cells seeded in 12-well plates. An overlay of DMEM-2 containing 0.05% methylcellulose was added to the cells and plates were incubated for 72 h at 37 °C. Plaques were visualized by staining cells with crystal violet supplemented with 10% formalin for 15 min.

### 2.5. Confocal Imaging

HEK293T and COG KO cells were seeded on glass bottom culture slides (Mattek Corporation, Ashland, MA, USA) and infected with VACV WR A4-YFP. After 24 h, cells were washed with 1 × PBS and fixed with 4% paraformaldehyde for 15 min [22] at room temperature (RT), followed by permeabilization with 0.1% Triton X-100 and 0.05% Tween-20 in PBS buffer for 30 min. Cells were stained with Alexa Flour 647 Phalloidin (ThermoFisher) for actin visualization and with 4’,6-diamidino-2-phenylindole (DAPI) for nuclei visualization. After staining, cells were mounted with Prolong Antifade Mounting media (Molecular Probes) and imaged using the LSM 710 inverted confocal microscope (Zeiss, Oberkochen, Germany). Approximately 11–18 cells were imaged for each cell type, and the number of actin tails in each cell were counted and analyzed for statistical significance.

### 2.6. Luciferase-Based Entry Assay

Cells were incubated with VACV WR-LUC virus for 1 h at RT, then washed three times with 1 × PBS to remove unbound virus. Culture media was replenished, and the cells were incubated at 37 °C for an additional 1.5–6 h. For the EV-specific entry assays, BSC-40 cells were infected with VACV IHDJ-LUC virus in the presence of anti-L1 7D11 mouse monoclonal antibody (provided by USAMRIID) for 30 h. The supernatant was subsequently collected and added to COG KO cells. Luciferase activity was measured using the Luciferase Assay System (Promega, Madison, WI, USA) according to manufacturer’s instructions. In brief, cells were harvested and lysed with Reporter Lysis Buffer. The cell lysate was mixed with the Luciferase Assay Reagent and luciferase activity was measured using an ENSPIRE plate reader (PerkinElmer, Waltham, MA, USA).

### 2.7. Membrane Fusion Assay

Levels of viral fusion were measured by infecting WT, COG KO and rescued cells with VACV WR virus labeled with a lipophilic tracer as previously described [28]. Specifically, VACV WR virus was labeled in the dark with DiD (Thermofisher) in 1 × PBS for 20 min at RT, followed by washing and pelleting to remove excess DiD. Cells were incubated with DiD labeled virus at 4 °C for 1 h in order to allow virus attachment, and then washed with 1 × PBS three times to remove unbound virus. The samples were placed at 37 °C or 4 °C for 90 min, and then fixed with 4% paraformaldehyde for 15 min. Following fixation, samples were analyzed by flow cytometry using the Attune Nxt instrument to determine percent DiD-positive cells.

### 2.8. Statistical Analysis

Biological replicates were performed in each experiment (as noted) and mean ± standard error is shown. Data were processed and analyzed using GraphPad Prism v7 (GraphPad Software, version 7, San Diego, CA, USA). Significance was reported in terms of adjusted *p* value, calculated using a one-way ANOVA and the Dunnett’s test for multiple comparisons.

## 3. Results

### 3.1. Reduced Size of MPXV Foci in COG KO Cells

**Figure 1:**
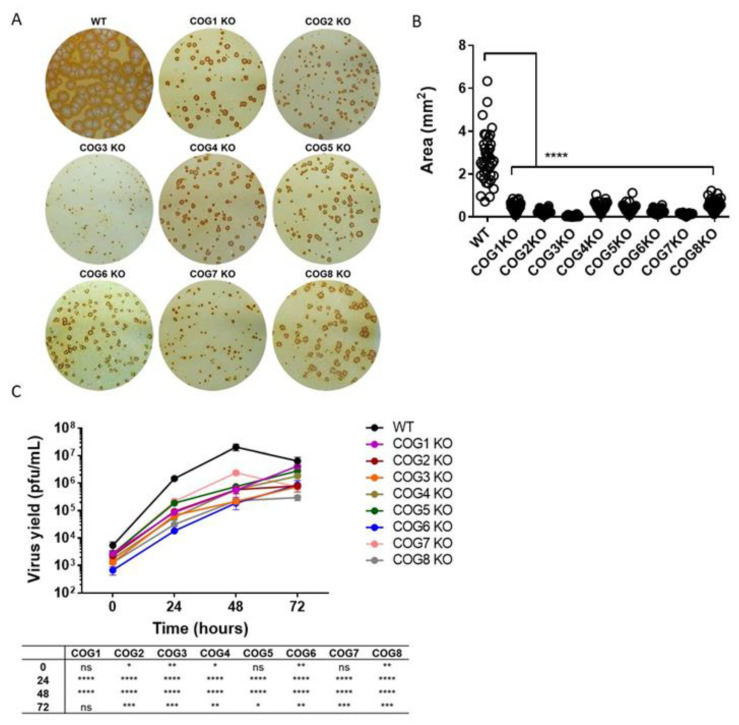
Decreased virus spread and yield in absence of conserved oligomeric Golgi COG genes. (**A**) Immunostaining of COG subunit knockout (KO) and wild type (WT) cells infected with West African monkey virus (MPXV WA) for 72 h. The infected cell monolayers were stained using an anti-vaccinia virus (VACV (c7D11 antibody, followed by an HRP-conjugated secondary antibody, and developed using o-dianisidine solution; (**B**) Area of individual foci in KO cell lines compared to WT. A total of 40 foci from each cell type were analyzed to determine area, and the average +/− standard error of the mean (SEM) are shown. Statistical difference between WT and KO cell lines indicated with asterisks; (**C**) Multi-step growth curve of MPXV WA in COG KO and WT cells. Virus yields determined at 0, 24, 48, and 72 hpi by plaque assay with 2–3 replicates per condition. Plotted values represent mean +/− SEM. Statistical difference between WT and KO cells for each time point tabulated below graph. For both (**B**) and (**C**), asterisks denoting specific *p* values. ns = not significant, * *p* ≤ 0.05, ** *p* ≤ 0.01, *** *p* ≤ 0.001, **** *p* < 0.0001.

Our previous haploid genetic screen using HAP1 cells infected with MPXV identified 48 candidate genes important for viral infection, including COG complex genes [16]. The COG complex is composed of two associated subcomplexes or lobes. Lobe A consists of COG1–4 and Lobe B consists of COG5–8. Since genes from both lobes were significantly enriched in the previous haploid screen, we further investigated the role of all COG complex genes in OPXV infection. WT HEK293T cells and cells lacking individual COG subunits were infected with MPXV WA and virus spread was evaluated by virus immunostaining (Figure 1A). Viral foci in each COG KO cell line appeared smaller compared to WT, suggesting that each COG subunit is important for viral infection. Notably, the extent of the decrease in focus size varied between cell lines, suggesting that some COG subunits were more critical for infection than others (Figure 1A). To confirm the statistical significance of this phenotype, we measured the area of 40 foci from each KO cell type and compared these values to WT. WT foci, on average, were significantly larger compared to all 8 KO cell types (Figure 1B).

### 3.2. Reduced MPXV Yield in COG KO Cells

To determine whether the COG complex genes are important for virus production, a multi-step infection was performed. COG subunit KO cells were infected with MPXV WA at a low MOI (0.01) for 72 h. Total virus yields at different times post infection (0, 24, 48 and 72 h) were determined by plaque assay (Figure 1C). We observed that all 8 KO cell lines produced significantly less virus than WT at 24 h and 48 h post infection. At 72 h, virus yields for 7 of the 8 KO cell lines were significantly lower than WT. These data corroborate our virus immunostaining results (Figure 1A) and show that the absence of COG subunits can impair virus production.

### 3.3. VACV Entry Reduced in COG KO Cells

**Figure 2:**
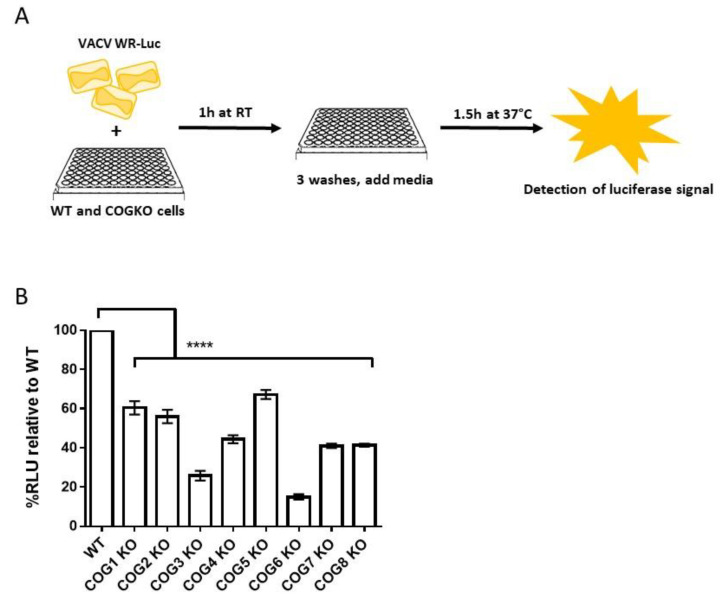
Lower virus entry in cells lacking COG genes. (**A**) Schematic representation of the luciferase-based viral entry assay; (**B**) Percent luciferase signal relative to WT in WT and COG KO cells infected with VACV-WR-LUC. Bars represent the mean of 3 replicates and error bars represent standard error. All groups compared to WT using a one-way ANOVA and Dunnett’s multiple comparison test: **** *p* < 0.0001.

Viral entry specifically refers to the step in which the viral core enters the cytoplasm, which occurs after virus attachment and fusion. To measure entry, cells were infected with VACV WR-LUC, which contains a firefly luciferase cassette expressed from a synthetic early/late promoter (Figure 2A). The detection of luciferase signal represents early viral gene expression which was used as a surrogate for viral entry. The relative luminescence units (RLU), a measure of luciferase activity, were significantly reduced in all infected COG KO cells compared to WT cells (Figure 2B). The greatest decrease in luciferase expression was observed in COG3 KO and COG6 KO cells (Figure 2B).

### 3.4. COG Complex Plays a Role in VACV Egress

**Figure 3:**
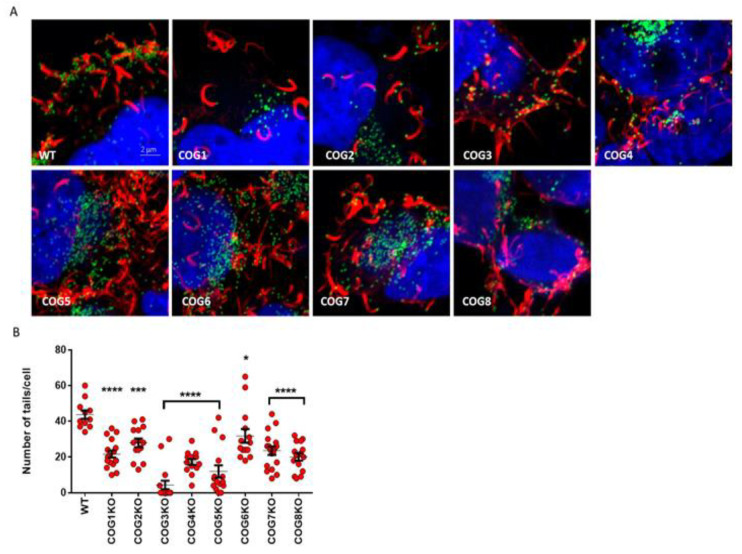
Fewer actin tails in COG KO cells compared to WT. (**A**) Confocal images of COG KO and WT cells infected with VACV A4-YFP for 24 h. Viruses are identified by the green dots, actin tails are visualized with Alexa Flour 647 phalloidin (red), and nucleus is visualized with DAPI (blue). Original magnification 40×. Approximately 11–18 cells were imaged per condition, and one representative image is shown per cell type; (**B**) Scatter plot showing the number of actin tails per cell in each treatment. Actin tails in cells from each treatment group were enumerated (*n* = 11–18 cells per cell type). The average number of actin tails per cell and SEM have been shown. All groups compared to WT using a one-way ANOVA and Dunnett’s multiple comparison test: * *p* ≤ 0.05, *** *p* ≤ 0.001, **** *p* < 0.0001.

EV release from a host cell can occur through a variety of mechanisms including exocytosis, budding and actin tail formation. After actin tail polymerization takes place, cell-associated EVs can be loaded onto protrusions that extend to neighboring cells for cell-to-cell spread [13,29]. To determine if the defect in COG complex proteins affected EV release like GARP complex proteins, we infected WT and KO cells with VACV-A4-YFP and imaged cell-associated EV by confocal microscopy. As shown in Figure 3A, we observed actin tails (filaments in red) in all COG KO infected cells. However, there was a clear difference in the number of actin tails imaged per cell (Figure 3B). Actin tails were the most abundant in WT cells, present at significantly higher numbers than in the KO cells. Of note, we observed few to no actin tails in COG3 KO cells. Together, these data demonstrate that the lack of COG genes could impair EV release, and consequently viral spread, by partially inhibiting actin tail formation.

### 3.5. Ectopic Expression of COG4 and COG7 Restores Virus Spread

**Figure 4:**
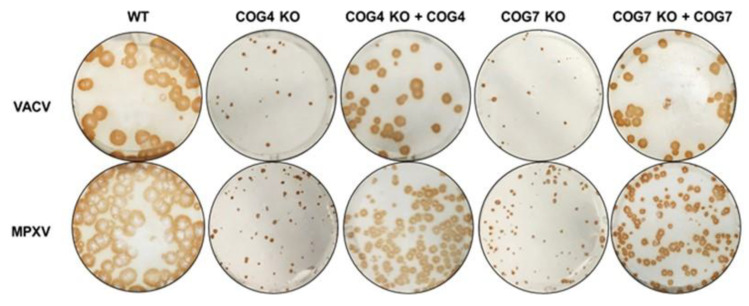
VACV and MPXV spread affected by COG4 and COG7 expression. Immunostaining of WT, COG KO and rescued cells infected with VACV (**top** panel) and MPXV WA (**bottom** panel) for 72 h. The infected cell monolayers were stained using an anti-VACV antibody c7D11, followed by an HRP-conjugated secondary antibody, and developed using o-dianisidine solution.

To confirm the involvement of COG genes in the viral processes described earlier, we characterized OPXV infection in KO cells stably transfected with the missing COG genes. As shown in Figure 4, we infected COG4 KO and COG7 KO cells and their respective rescued cell lines with VACV and MPXV and visualized viral spread by immunostaining. Concordant with the data shown in Figure 1A,B, cells lacking COG4 or COG7 genes had smaller foci compared to WT cells. However, the addition of the missing COG gene through complementation clearly increased the size of VACV and MPXV foci (Figure 4). For both viruses, rescued cells produced foci intermediate in size between the KO and WT cells. These comparisons between KO and rescued cells confirmed that viral spread is dependent on COG complex genes and that this phenotype is not an off-target effect of the CRISPR methodology used to generate the KO cells.

### 3.6. Entry of MV and EV VACV Particles is Reduced in Cells Lacking COG4 and COG7

**Figure 5:**
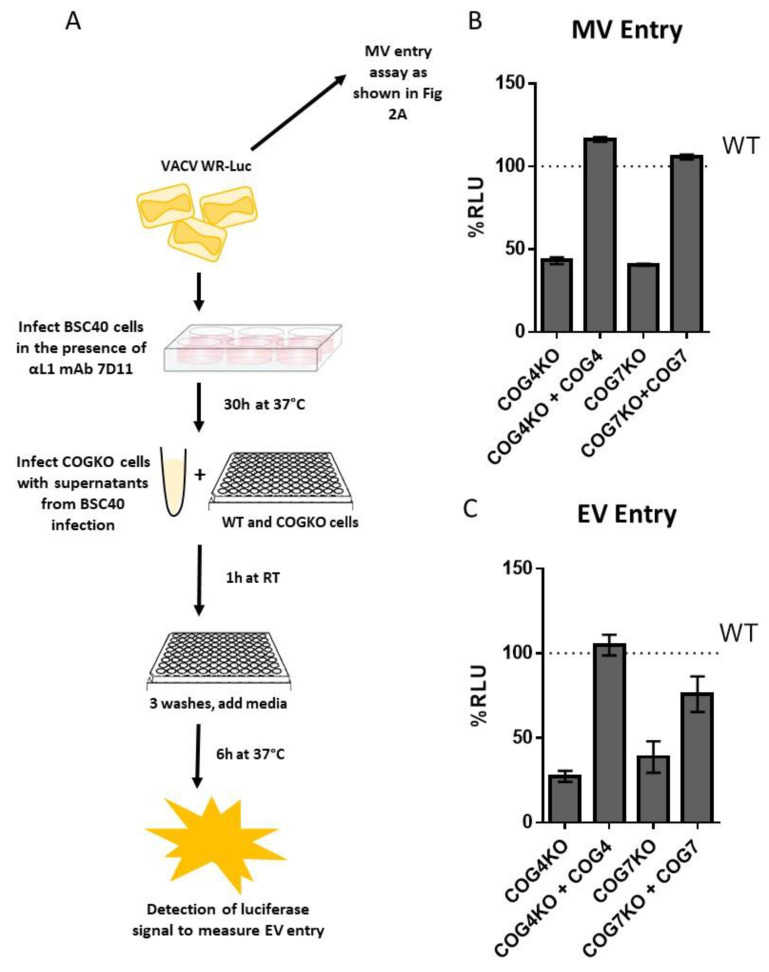
Reduced EV and MV entry in COG-deficient cells. (**A**) Schematic showing steps to quantitate MV and EV entry; (**B**) Percent RLU relative to WT for MV entry for WT, COG KO and COG rescued cells; (**C**) Percent RLU relative to WT for EV specific entry for WT, COG KO and COG rescued cells. For both MV and EV luciferase entry assays, each treatment was tested in triplicate, and the mean of *n* = 3 is shown. Error bars represent standard error.

Viral entry can occur through both the MV and EV forms of OPXV particles. In order to evaluate entry of MV and EV separately, WT, KO and rescued cells were infected with VACV-LUC, harvested from infected cells (mainly MV) and from supernatant (mainly EV). To selectively quantitate entry of EV particles, BSC40 cells were infected in the presence of an anti-L1 antibody 7D11, an antibody known to potently neutralize MV particles by binding to the L1 protein expressed on the MV surface. The MV-depleted supernatants were then used to infect WT, COGKO and rescued cells to measure EV entry (Figure 5A). As shown in Figure 5B,C, both MV and EV entry were noticeably reduced in the KO cell lines compared to WT cells. Additionally, luciferase levels were restored to WT levels in the rescued cells demonstrating the specific involvement of COG4 and COG7 genes in the reduction of EV and MV entry (Figure 5B,C).

### 3.7. COG4 and COG7 are Important for Viral Fusion

**Figure 6:**
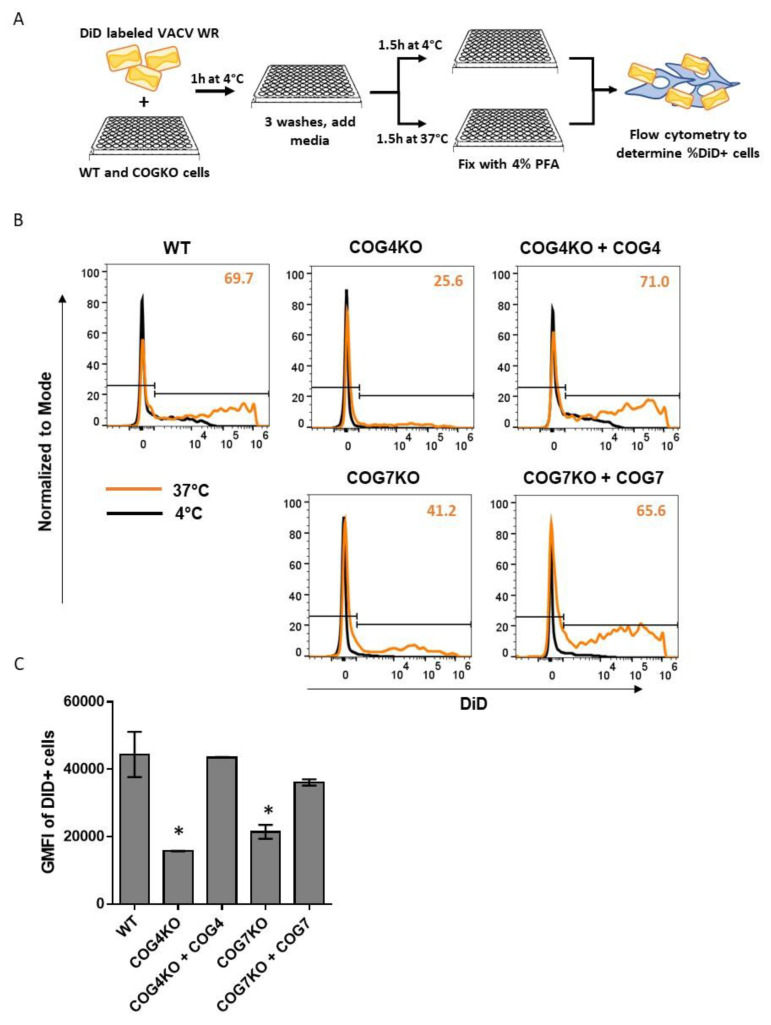
COG genes impact VACV fusion with host cell. (**A**) Schematic representation of membrane fusion assay using DiD-labeled VACV WR strain; (**B**) Histograms showing baseline fluorescence at 4 °C (black) and shift due to fusion at 37 °C (orange) for WT, KO, and rescued cell lines incubated with DiD labeled virus. Histograms normalized to mode (y-axis). X-axis denotes intensity of DiD fluorescence. The percentage of DiD-positive cells at 37 °C shown in the top right corner of each histogram plot in orange. Each treatment was tested in duplicate and one representative histogram overlay has been shown per condition; (**C**) Bar graph comparing the geometric mean fluorescence intensity (GMFI) of the DiD-positive population at 37 °C in each cell type. Values shown represent the average of two replicates +/− SEM. Statistical significance determined based upon one-way ANOVA and Dunnett’s multiple comparisons test. * *p* ≤ 0.05.

To determine the role of COG subunits in the viral fusion step, WT, COG KO and rescued cells were infected with VACV labeled with fluorescent probe DiD. Virus and cells were first incubated together at 4 °C to facilitate viral attachment but not entry. Unbound virus was then washed away, and the virus-bound cells were incubated at either 37 °C (to allow viral fusion) or at 4 °C (to measure the background signal) (Figure 6A). As shown in Figure 6B, the DiD-positive populations at 37 °C (orange histograms) were lower for the KO cell types compared to WT or rescued cells. Viral fusion, as indicated by the frequency of DiD-positive cells at 37 °C, was much lower in COG4KO (25.6%) and COG7KO (41.2%) cells compared to WT (69.7%) (Figure 6B). In both rescued cell types, the percentages of DiD-positive cells were restored to WT levels (71% and 65.6% for COG4KO+COG4 and COG7KO+COG7, respectively). Additionally, the geometric mean fluorescent intensity (GMFI) of the DiD-positive populations in the 5 cell types exhibited the same pattern. Namely, the GMFI of DiD-positive cells for COG4KO and CO7KO were significantly lower than those for WT and rescued cells (Figure 6C). Taken together, these data clearly show that the COG complex, specifically COG4 and COG7, are important for viral fusion.

## 4. Discussion

Poxvirus infection is a complex process that involves several virus-encoded proteins as well as host proteins and pathways. Numerous independent genome-wide genetic screens have been performed to date which have revealed a variety of host genes and pathways that may be required for poxvirus infection. These include genes that constitute the GARP and COG complexes, two different vesicle tethering complexes that function at the Golgi apparatus [14,16,17]. The GARP complex is a multi-subunit tethering complex that is important for retrograde transport, a process that involves membrane fusion of endosome-derived vesicles at the trans-Golgi network [30]. The importance of the retrograde pathway and GARP complex genes for proper EV membrane formation and viral cell-to cell spread has been previously shown [16,17,31]. The COG complex, on the other hand, plays a crucial role in membrane trafficking and Golgi homeostasis by regulating glycosylation enzymes, intra-Golgi retrograde vesicle trafficking, and maintaining Golgi structure, in addition to its involvement in endosome to Golgi transport [18,19,20]. The COG complex is comprised of two associated subcomplexes or lobes, each with four respective subunits, COG1–4 and COG5–8 [21]. Given the similarities in structural composition and functions, we hypothesized that the COG complex may also play a role in poxvirus infection. In this study, we demonstrate the specific involvement of several subunits of the COG complex in OPXV fusion, entry and release in vitro.

We used cell lines containing stable knockouts of all 8 individual subunits (COG1–8) as a tool to study the effects of each COG subunit on OPXV infection. By immunostaining virus infected COG1–8 KO cells, we showed that the absence of each individual subunit decreased the size of viral foci, suggesting that every subunit has an impact on viral infection. This effect was further demonstrated in multistep infections showing decreased viral levels in infected COG subunit KO cells compared to WT over time. Viral yields were significantly decreased at 24 and 48 hpi for all 8 KO cell types, and at 72 h for all but the COG1 KO cell line. The lower levels of viral spread could be attributable to defects in viral egress. Although actin tails were visualized in all 8 KO cell types, their frequency was significantly lower in KO cells compared to WT. The presence of actin tails and viral plaques in all 8 KO cell types suggests that while none of the COG genes are essential for viral spread individually, all 8 genes partially impact viral egress and dissemination.

The entry of OPXVs into host cells occurs at the plasma membrane or through endocytic pathways [32,33]. The entry process is unique in that it requires a large number of viral proteins for attachment, fusion and core release. We demonstrated that the COG complex impacts the entry of both MV and EV viral particles using a luciferase-based quantification method. Luciferase signal, indicative of early gene expression, was significantly lower in all 8 KO cells lines compared to WT signifying lower levels of viral entry. Luciferase levels were restored to WT values in COG4- and COG7 rescued cell lines, showing a definitive link between the KO of COG genes and reduced viral entry. Additionally, we determined whether COG genes had any effect on viral fusion by infecting WT, KO and rescued cells with DiD-labeled VACV. We observed evidence of lower viral fusion in KO cells, and that the reduced fusion phenotype was restored in rescued cells.

Other pathogens, both viral and bacterial, also exploit pathways regulated by the COG complex for replication and pathogenesis. For HIV-1, the COG complex is important for early viral life cycle steps that are involved in replication but not fusion [22]. On the other hand, by redirecting Golgi-derived vesicles, *Chlamydia* and *Brucella* exploit the COG complex to promote bacterial biogenesis and replication [23,24]. Although our data suggest that deficiencies in the COG complex lead to impaired viral entry and reduced spread, it would be worthwhile to further investigate if OPXVs utilize COG complex trafficking pathways for infection. Additionally, although the COG complex functions as a unit, single subunit knockouts can destabilize other subunits and the complex as a whole [20]. We observed this in effect in our study, where single gene knockouts resulted in the impairment of several viral processes albeit to varying degrees. Notably, the absence of single COG genes did not completely block viral infection either, suggesting some overlap in the functions of the different COG proteins, or possibly redundancies with other proteins or complexes not identified in this study.

The identification of host pathways for viral infection has revealed several novel targets for therapeutic intervention, including the retrograde transport pathway. In this study, we have further validated the role of the COG complex in viral entry, fusion and egress. Although the impact of each individual subunit varied depending upon the specific viral process in question, overall there was a substantial decrease in viral entry, actin tail formation, and ultimately viral spread in the absence of COG genes. The poxvirus life cycle is a complex multistep process that involves a multitude of host proteins for successful viral infection. The data presented in this study demonstrate that the genes of the COG complex play a significant role in the OPXV life cycle.
